# Unveiling the role of coagulation-related genes in acute myeloid leukemia prognosis and immune microenvironment through machine learning

**DOI:** 10.1186/s40001-025-02975-9

**Published:** 2025-08-11

**Authors:** Liyun Ji, Yanxia Yang, Siyue Ma

**Affiliations:** 1 Department of Hematology, The Affiliated Cancer Hospital of Zhengzhou University, Henan Cancer Hospital, Zhengzhou, 450008 Henan China; 2 Department of General Surgery, The Affiliated Cancer Hospital of Zhengzhou University, Henan Cancer Hospital, Zhengzhou, 450008 Henan China

**Keywords:** Acute Myeloid Leukemia, Coagulation, Consensus Clustering Analysis, Prognostic Model, Immune Microenvironment

## Abstract

**Background:**

Acute Myeloid Leukemia (AML) is a highly heterogeneous hematologic malignancy influenced by various factors affecting prognosis. Recently, the role of coagulation-related genes in tumor biology has garnered increasing attention. This study aims to investigate the expression patterns of coagulation-related genes in AML and their clinical relevance.

**Methods:**

We obtained RNA-seq data and clinical information for AML patients from The Cancer Genome Atlas (TCGA) and the Gene Expression Omnibus (GEO), followed by data cleaning and normalization. Unsupervised consensus clustering was performed to identify molecular subtypes, and Kaplan–Meier survival analysis was utilized to assess survival differences. We further identified differentially expressed genes (DEGs) between groups and conducted functional enrichment analyses. Additionally, a prognostic model was constructed using machine learning techniques, and its prognostic ability was validated.

**Results:**

Clustering analysis categorized 151 tumor samples into the high coagulation-related gene expression group (C1, high-expression) and the low coagulation-related gene expression group (C2, low-expression), revealing 1,747 DEGs. Functional enrichment analysis indicated that DEGs were mainly associated with leukocyte migration and cytokine signaling pathways. Immune landscape analysis showed that the high expression group had elevated immune and stromal scores, distinct immune cell infiltration patterns, and a higher ESTIMATE score. The constructed coagulation score risk model indicated that age, cytogenetics, and risk scores were significantly associated with AML prognosis. Furthermore, intersection analysis using three machine learning methods identified MMP7 and F12 as key biomarkers.

**Conclusion:**

Our study demonstrates that coagulation-related genes play a crucial role in the molecular characteristics, prognostic assessment, and immune modulation in AML. MMP7 and F12 are highlighted as potential biomarkers that could aid in optimizing the diagnosis and treatment strategies for AML. These findings offer new insights into personalized therapies for AML.

## Introduction

Acute myeloid leukemia (AML) is a highly aggressive hematological malignancy characterized by the abnormal proliferation and impaired differentiation of myeloid precursor cells, leading to the accumulation of immature leukemic cells in the bone marrow and peripheral blood [1]. Prognosis varies significantly with age, highlighting stark disparities in survival rates: only 9% of individuals aged 65 and older survive five years, compared to 35% for those aged 50–64 and 58% for patients aged 20–49 [2]. Although chemotherapy and hematopoietic stem cell transplantation are cornerstone treatments, recent advances in molecularly targeted therapies-such as FLT3 inhibitors and IDH inhibitors-have markedly improved response rates in select populations [3–5]. Nevertheless, many patients experience short remission periods followed by disease relapse, underscoring the urgent need for more effective prognostic and therapeutic strategies.

Cytogenetic and molecular abnormalities play pivotal roles in determining the responsiveness of leukemia cells to treatment, ultimately influencing patient outcomes. The European Leukemia Network (ELN) established risk stratification criteria in 2017 based on these abnormalities; however, these criteria do not fully account for gene expression profiles critical to the pathogenesis of AML [6]. Despite widespread acceptance of the ELN 2017 risk stratification as a prognostic standard, it often inadequately predicts outcomes for a substantial proportion of AML patients, highlighting the pressing need for a more nuanced classification system that integrates molecular and genetic insights.

In addition, coagulation dysfunction has emerged as a significant complication in AML, greatly impacting survival and morbidity rates. This dysfunction complicates chemotherapy administration, presenting considerable challenges in patient management. Recent studies indicate that patients with hematological malignancies exhibit an elevated risk of venous thromboembolism (VTE) and arterial thromboembolism (ATE), with incidence rates comparable to those seen in solid tumor patients [7–10]. Mechanisms underlying this increased thrombotic risk include aberrant activation of the coagulation cascade and platelet aggregation, both critical in mediating thrombosis. Notably, in various cancers, coagulation processes are intricately linked to tumor progression rather than merely secondary consequences. The tumor microenvironment (TME) significantly influences cancer growth, migration, and treatment resistance. Recent studies indicate that malignant tumors promote coagulation pathways by secreting procoagulants such as tissue factor (TF, encoded by the F3 gene), leading to a hypercoagulable state linked to VTE formation, localized hypoxia, and subsequent remodeling of the tumor microenvironment, which supports tumor progression and metastasis [11–13]. Moreover, platelets release growth factors, cytokines, and coagulation factors that can suppress immune functions, including those of natural killer (NK) cells and T cells, facilitating immune evasion and tumor progression [14]. Graf et al. [15] demonstrated that coagulation factor FX, produced by myeloid cells, contributes to immune evasion; its inhibition enhances dendritic cell and cytotoxic T cell presence in tumors and improves anti-tumor immunity when combined with Anti-PD-L1 (anti-programmed death-ligand 1) therapy.

In this study, we utilized consensus clustering analysis to stratify AML samples into two distinct groups based on their molecular profiles, specifically focusing on coagulation-related genes. This analysis revealed significant differences in overall survival rates and immune cell infiltration levels between the groups, providing novel insights into how coagulation-related gene expression impacts AML prognosis. The findings suggest potential pathways for enhancing early diagnosis and informing immunotherapeutic strategies in AML.

## Results

### DEGs identification and coagulation-related tumor cluster classification

**Fig. 1:**
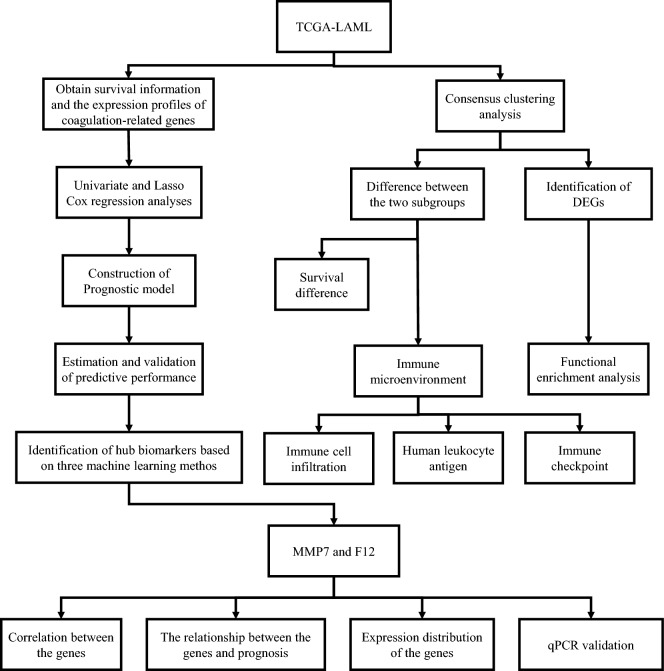
Workflow Diagram of the Current Study

**Fig. 2:**
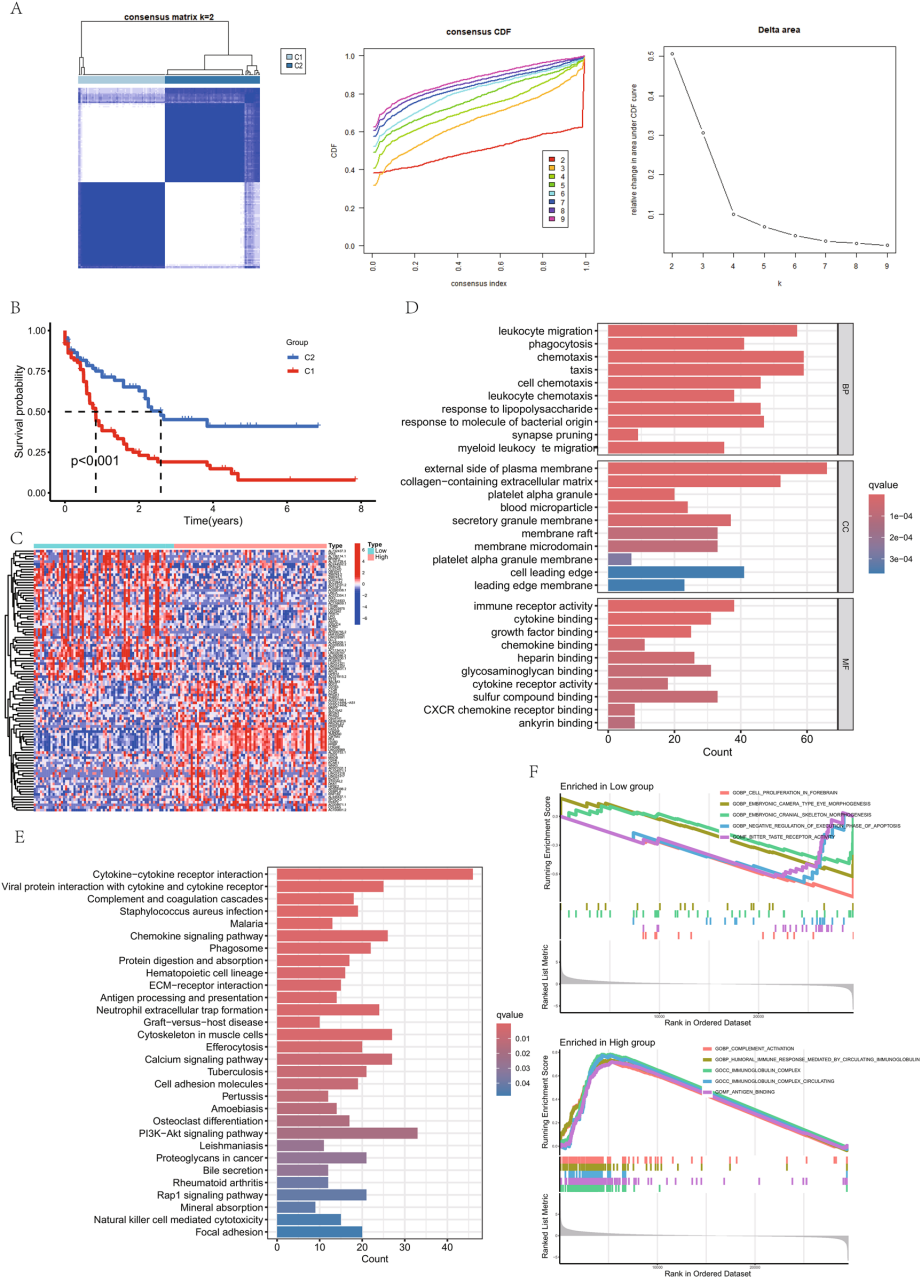
Stratification of AML Patients Based on Coagulation-Related Gene Expression Profiles. **A** 151 TCGA-LAML samples were clustered into two groups (C1 and C2) based on the expression of 138 coagulation-related genes, showing molecular heterogeneity. **B** Survival analysis reveals significant differences between the two clusters, highlighting the prognostic value of coagulation-related gene expression. **C** Heatmap shows distinct gene expression profiles between clusters. **D** Gene Ontology (GO) enrichment analysis shows that differentially expressed genes are associated with processes like leukocyte migration and immune response, indicating potential roles in AML. **E** Kyoto Encyclopedia of Genes and Genomes (KEGG) pathway analysis highlights enrichment in AML-related pathways, including cytokine signaling. **F** Gene Set Enrichment Analysis further reveals pathways and functions associated with each cluster, emphasizing the biological relevance of coagulation-related gene expression in AML

Figure 1 provides an overview of the study flow. Cluster analysis of 151 tumor samples from the TCGA cohort, based on the expression levels of 138 coagulation-related genes, was performed. This analysis, with optimal clustering at *k* = 2, classified the samples into two distinct groups: the high coagulation-related gene expression group (C1, high-expression) and the low coagulation-related gene expression group (C2, low-expression) (Fig. 2A). Kaplan–Meier survival analysis showed that the coagulation high group had a significantly worse prognosis compared to the coagulation low group (Fig. 2B). In comparing these groups, we identified 1747 differentially expressed genes, of which 1422 were up-regulated and 325 were down-regulated, applying a |logFC|> 1 and a *p*-value <0.05 as screening thresholds. These expression differences were depicted in a heatmap (Fig. 2C).

### Functional enrichment analysis between high and low coagulation groups

We analyzed the differentially expressed genes between the high and low coagulation groups and performed functional enrichment analyses utilizing Gene Ontology (GO), Kyoto Encyclopedia of Genes and Genomes (KEGG), and Gene Set Enrichment Analysis (GSEA).

GO analysis revealed several key pathways in different categories. Under Biological Process, significant pathways included the leukocyte migration, phagocytosis, chemotaxis, taxis, cell chemotaxis, leukocyte chemotaxis, response to lipopolysaccharide, response to molecule of bacterial origin, synapse pruning, and myeloid leukocyte migration (Fig. 2D). For Cellular Component, the analysis highlighted pathways such as external side of plasma membrane, collagen-containing extracellular matrix, platelet alpha granule, blood microparticle, secretory granule membrane, membrane raft, membrane microdomain, platelet alpha granule membrane, cell leading edge, and leading edge membrane (Fig. 2D). In Molecular Function, prominent pathways included immune receptor activity, cytokine binding, growth factor binding, chemokine binding, heparin binding, glycosaminoglycan binding, cytokine receptor activity, sulfur compound binding, CXCR (C-X-C chemokine receptor) chemokine receptor binding, and ankyrin binding (Fig. 2D).

KEGG pathway analysis identified several critical pathways impacted by the differentially expressed genes, such as Cytokine-cytokine receptor interaction, Viral protein interaction with cytokine and cytokine receptor, Complement and coagulation cascades, Staphylococcus aureus infection, Malaria, Chemokine signaling pathway, Phagosome, Protein digestion and absorption, Hematopoietic cell lineage, and ECM-receptor interaction (Extracellular Matrix-receptor interaction) (Fig. 2E). These results underscore the significant role these genes play in various cellular and molecular processes.

GSEA further revealed that the coagulation low group showed positive associations with cell proliferation in forebrain, embryonic camera type eye morphogenesis, embryonic cranial skeleton morphogenesis, negative regulation of execution phase of apoptosis, and bitter taste receptor activity (Fig. 2F). Conversely, the coagulation high group was enriched in pathways related to complement activation, humoral immune response mediated by circulating immunoglobulin, immunoglobulin complex, immunoglobulin complex circulating, and antigen binding (Fig. 2F).

This thorough analysis using GO, KEGG, and GSEA provides a comprehensive view of the functional pathways affected by the differentially expressed genes, enhancing our understanding of their biological roles and implications.

### Immune landscape analysis between coagulation high and low groups

**Fig. 3:**
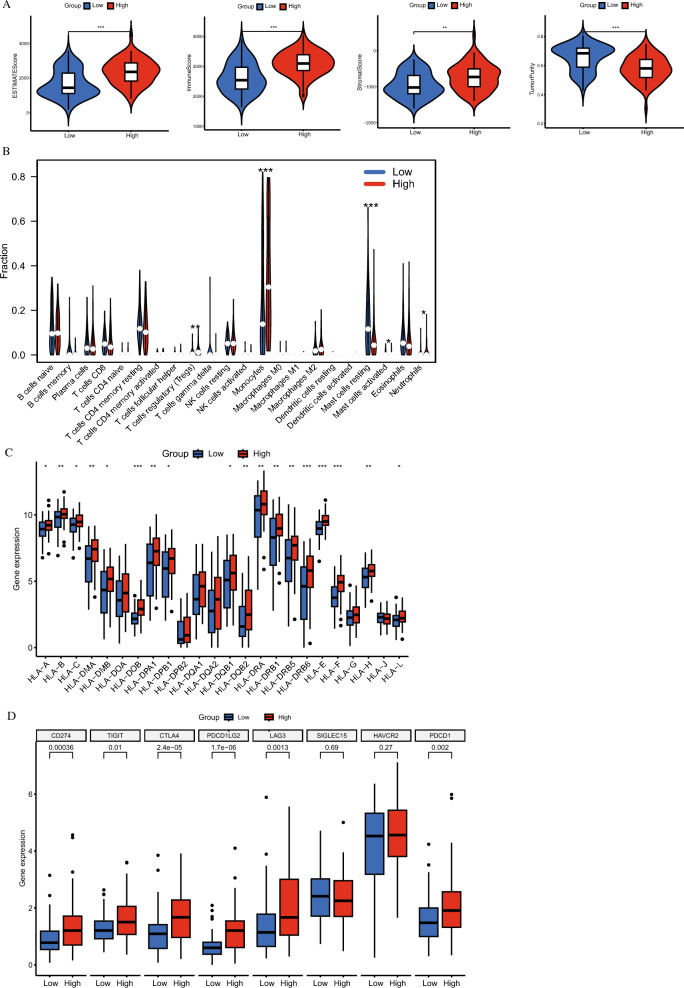
Immune Microenvironment Differences Between High and Low Clusters in AML. **A** A box plot comparing the differences in ESTIMATE score, immune score, stromal score, and tumor purity between the high and low clusters, highlighting distinct immune microenvironment characteristics. **B** The difference in the infiltration of 22 types of immune cells between the two clusters, revealing varied immune profiles. **C** Differential expression of human leukocyte antigen (HLA) genes between the high and low clusters, suggesting differences in immune recognition and response. **D** The differential expression of immune checkpoint genes between the two clusters, providing insights into immune evasion mechanisms in AML

The ESTIMATE algorithm, which stands for Estimation of STromal and Immune cells in MAlignant Tumor tissues using Expression data, was specifically utilized in this study to comprehensively evaluate the presence and proportions of immune and stromal components within the tumor microenvironment. The analysis revealed that the coagulation high group had elevated immune scores, stromal scores, and ESTIMATE scores, while the coagulation low group demonstrated lower tumor purity (Fig. 3A).

The comparative analysis indicated that the coagulation high group exhibited significantly reduced infiltration levels of both resting and activated mast cells. In contrast, this group showed increased infiltration levels of regulatory T cells (Tregs), monocytes, and neutrophils compared to the coagulation low group (Fig. 3B). Further examination of human leukocyte antigen (HLA) and immune checkpoint expression levels between the two groups is presented in Fig. 3C and D.

These observations highlight the substantial impact of coagulation-related genes on the immune landscape of the tumor microenvironment, revealing distinct immunological profiles associated with high and low expression of coagulation-related genes.

### Construction of genetic risk model

**Fig. 4:**
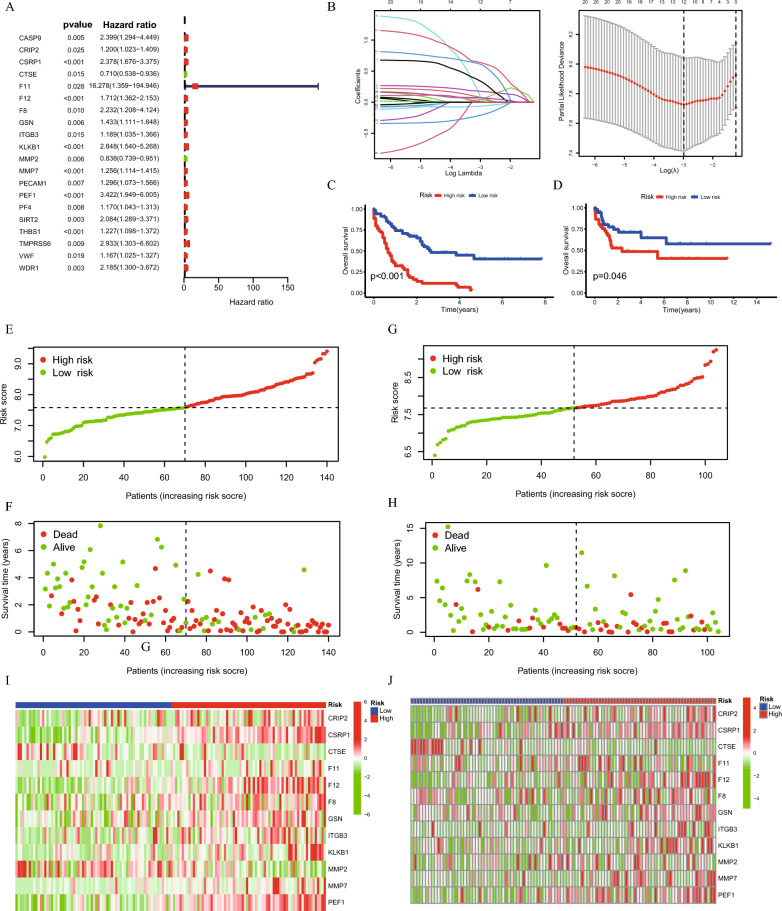
Prognostic Model Development and Survival Analysis Based on Coagulation-Related Genes in AML. **A** Univariate Cox regression identifies 20 genes significantly associated with the prognosis of AML patients, providing a foundation for further model development. **B** Lasso Cox regression analysis selects 12 genes to construct a prognostic model, termed the coagulation risk score, to predict AML patient outcomes. **C** and **D** Kaplan-Meier survival plots demonstrate significant survival differences between high- and low-risk groups based on the coagulation risk score, validated using TCGA and GEO datasets. **E** and **F** The distribution and relationship between survival prognosis and risk score are shown for the TCGA dataset, further supporting the prognostic value of the model. **G** and **H** Similar analysis of survival prognosis and risk score distribution is performed for the GEO dataset, confirming the robustness of the model. **I** and **J** Heatmaps display the expression patterns of the 12 genes between high- and low-risk groups in the TCGA and GEO datasets, respectively, highlighting the distinct molecular profiles associated with prognosis

The results of the univariate Cox regression analysis were illustrated using a forest plot, which highlighted 20 genes with significant prognostic associations in AML (Fig. 4A). To refine these findings, Lasso regression analysis was applied to reduce the number of genes and enhance the model’s specificity (Fig. 4B). This process led to the selection of 12 key genes for the development of the coagulation score risk model. Kaplan–Meier survival curves were then plotted to visualize the prognostic impact of the coagulation score model. Analysis indicated that the high-risk group had a poorer prognosis compared to the low-risk group in both the training and validation datasets (Fig. 4C and D). The risk score distribution plots further supported coagulation score as a reliable prognostic indicator for AML (Fig. 4E–H). Additionally, two separate heatmaps were used to display the expression patterns of these 12 genes between the high- and low-score groups in the TCGA and GEO datasets, respectively (Fig. 4I and J).

**Fig. 5:**
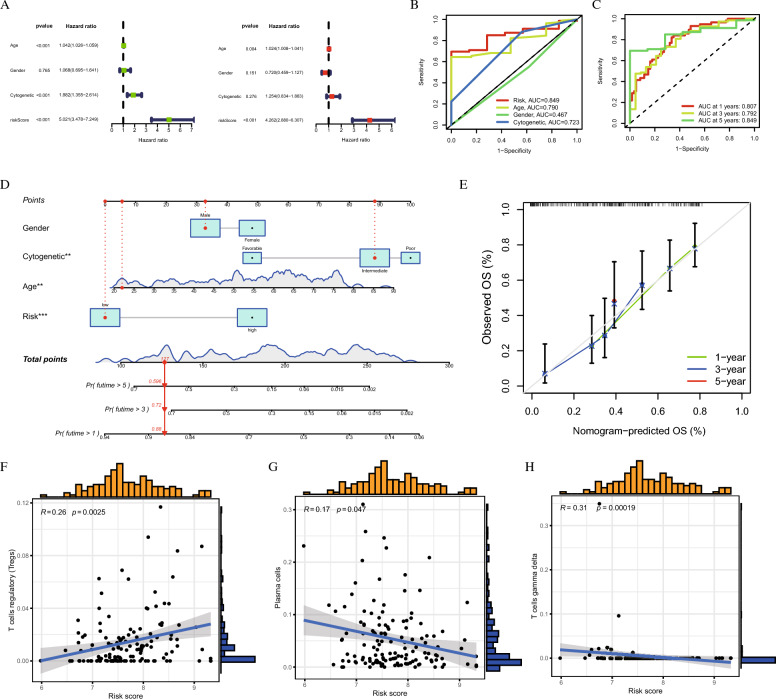
Identification of Hub Genes and Prognostic Model Evaluation Using Machine Learning in AML. **A** Univariate and multivariate Cox regression analyses identify independent prognostic factors for AML patients, revealing key variables associated with patient survival. **B** The ROC curve demonstrates the predictive performance of clinical features in distinguishing high-risk and low-risk AML patients, highlighting their prognostic value. **C** The ROC curve shows the predictive accuracy of the coagulation risk score, confirming its ability to stratify AML patients based on prognosis. **D** and **E** A nomogram and the corresponding C-index are presented to predict individual patient survival, incorporating the coagulation risk score and clinical features for more precise risk assessment. The correlation between the risk score and the infiltration of T regulatory cells (**F**), plasma cells (**G**), and gamma delta T cells (**H**) is shown, revealing the immune landscape’s influence on patient prognosis

Univariate regression analysis revealed that the Age, Cytogenetic, and the risk score were significantly associated with AML prognosis (Fig. 5A). Multivariate regression analysis confirmed that higher Age and elevated risk scores were indicative of worse prognosis (Fig. 5A). Figure 5B highlights the prognostic strength of clinical features in AML, showcasing Age (Area Under the Curve, AUC = 0.790), Cytogenetics (Area Under the Curve, AUC = 0.723), and Risk score (Area Under the Curve, AUC = 0.849). This ROC (Receiver Operating Characteristic) curve collectively demonstrates their effectiveness in predicting patient outcomes. Furthermore, Fig. 5C focuses on the risk score’s exceptional predictive capability for survival, revealing its accuracy for 1-year (Area Under the Curve, AUC = 0.807), 3-year (Area Under the Curve, AUC = 0.792), and 5-year (Area Under the Curve, AUC = 0.849) survival rates. Together, these figures delineate the complementary roles of clinical factors and risk score in the comprehensive assessment of AML prognosis.

Additionally, we developed a nomogram that integrated clinical features along with the coagulation risk score to evaluate its predictive performance (Fig. 5D). The concordance index (C-index) was calculated to assess the accuracy of the nomogram, as illustrated in Fig. 5E. Further correlation analysis showed a positive relationship between the risk score and regulatory T cells (Tregs) (*R* = 0.26, *p* = 0.0025) (Fig. 5F). On the other hand, the risk score was negatively correlated with plasma cells (*R* = −0.17, *p* = 0.047) (Fig. 5G), and gamma delta T cells (*R* = −0.31, *p* = 0.00019) (Fig. 5H). These findings suggest that the coagulation score is not only a significant prognostic marker but also has meaningful associations with specific immune cell populations, providing further insight into the underlying biological mechanisms in AML.

### Identification and functional analysis of hub biomarkers in AML

**Fig. 6:**
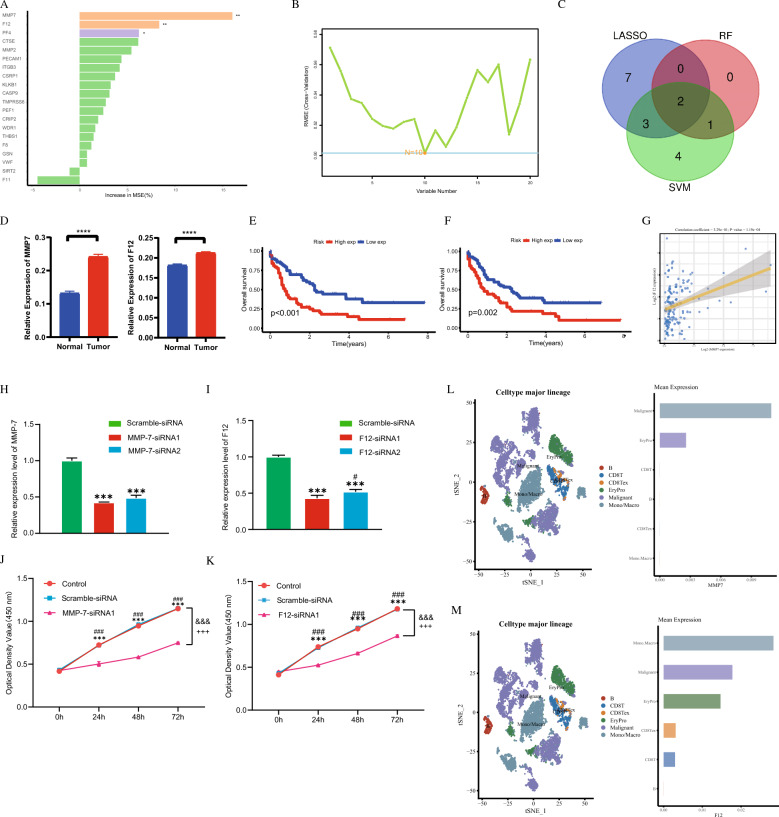
Identification of Hub Biomarkers and Prognostic Value in AML Using Machine Learning. **A** and **B** Random forest and support vector machine models are used to select genes associated with the prognosis of AML, identifying key prognostic markers. **C** A Venn plot illustrates the overlap of genes significantly associated with prognosis, highlighting a shared set of biomarkers identified by both machine learning methods. **D** qPCR validation confirmed the expression levels of MMP7 and F12. **E** and **F** Kaplan-Meier survival plots demonstrate the correlation between the expression of MMP7 and F12 and overall survival in AML patients, using data from the TCGA database, indicating their prognostic relevance. **G** The correlation between the expression levels of MMP7 and F12 in AML patients from the TCGA database is shown, suggesting a potential relationship between these biomarkers. **H** qPCR analysis showing mRNA expression levels of MMP-7 after transfection with siRNA1 or siRNA2 in SKM-1 cells. “Scramble-siRNA”: negative control; “MMP-7-siRNA1” and “MMP-7-siRNA2”: MMP-7 knockdown group. “***”: *P* < 0.001 vs. Scramble-siRNA. **I** qPCR analysis showing mRNA expression levels of F12 after transfection with siRNA1 or siRNA2 in SKM-1 cells. “Scramble-siRNA”: negative control; “F12-siRNA1” and “F12-siRNA1”: MMP-7 knockdown group. “***”: *P* < 0.001 vs. Scramble-siRNA; “#”: *P* < 0.05 vs. F12-siRNA1. **J** Bar graphs showing the proliferation activity of SKM-1 cells at 0, 24, 48, and 72 h after treatment. “Control”: untreated group; “Scramble-siRNA”: negative control; “MMP-7-siRNA1”: MMP-7 knockdown group. “###”: *P* < 0.001 vs. Control; “***”: *P* < 0.001 vs. Scramble-siRNA; “&&&”: *P* < 0.001 vs. Scramble-siRNA; “+++”: *P* < 0.001 vs. Control. **K** Bar graphs depicting the proliferation activity of SKM-1 cells at 0, 24, 48, and 72 h after treatment. “Control”: untreated group; “Scramble-siRNA”: negative control; “F12-siRNA1”: F12 knockdown group. “###”: *P* < 0.001 vs. Control; “***”: *P* < 0.001 vs. Scramble-siRNA; “&&&”: *P* < 0.001 vs. Scramble-siRNA; “+++”*: *P* < 0.001 vs. Control. **L** and **M** The differential expression of MMP7 and F12 in the AML immune microenvironment is analyzed based on data from GEO dataset, revealing how these biomarkers are modulated in the context of immune cell infiltration

To identify reliable biomarkers for prognosis and diagnosis, we conducted Random Forest and Support Vector Machine analyses in addition to Lasso regression. Random Forest identified 3 genes (Fig. 6A), while Support Vector Machine identified 10 genes (Fig. 6B). By taking the intersection of these three methods, we identified two genes, MMP7 and F12, as having the highest potential diagnostic and prognostic value (Fig. 6C). Additionally, qPCR validation of the clinical samples we collected revealed that both MMP7 and F12 were significantly upregulated in the bone marrow tissues of AML patients (Fig. 6D). Both genes were found to be highly expressed and associated with poorer prognosis, designating them as potential high-risk biomarkers for AML (Fig. 6E and F). The strong correlation observed between MMP7 and F12 suggests potential interactions that warrant further investigation (Fig. 6G).

To functionally validate these biomarkers, we performed targeted siRNA knockdown in SKM-1 cells, selecting optimal sequences through rigorous RT-qPCR validation (achieving >75% knockdown efficiency for both genes) (Fig. 6H and I). Subsequent proliferation assays demonstrated that suppression of either MMP7 or F12 significantly reduced SKM-1 cell viability (MMP7 KD: 58 ± 5% reduction at 72 h; F12 KD: 52 ± 4% reduction at 72 h; both *p* < 0.001 vs control) (Fig. 6J and K), confirming their critical roles in AML cell proliferation.

Single-cell RNA sequencing data from the GSE154109 dataset provided further insight into the expression patterns of these biomarkers. MMP7 was primarily expressed in Malignant Cells and Erythrocyte Progenitor (EryPro) Cells (Fig. 6L), while F12 was not only found in Malignant Cells and EryPro Cells, but also in various immune cell types, including Monocytes, Macrophages, CD8 + T cell exhaustion states, and CD8 + T cells (Fig. 6M).

These findings underscore the distinct roles of MMP7 and F12 in various cellular contexts, highlighting their potential significance in AML pathogenesis and providing promising avenues for further research and therapeutic targeting.

## Discussion

This study significantly advances our understanding of the prognostic landscape in Acute Myeloid Leukemia (AML) by developing a predictive model based on coagulation-related genes. The incorporation of a risk score derived from key biomarkers provides a novel tool for stratifying patients according to their prognosis. In an era where personalized medicine is paramount, such a model can assist clinicians in making informed treatment decisions, potentially leading to improved patient outcomes. Previous studies have demonstrated the utility of similar prognostic models in various malignancies, showing that integrating molecular data can enhance predictive accuracy and guide therapeutic strategies [16–19].

The integration of our predictive model into clinical practice holds transformative potential for patient management in Acute Myeloid Leukemia (AML). By employing the risk score derived from coagulation-related genes, clinicians can stratify patients into distinct prognostic categories, enabling a more nuanced approach to treatment. High-risk patients may benefit from aggressive strategies such as early intervention with intensive chemotherapy, targeted therapies, or even participation in clinical trials, with close monitoring of disease progression to allow for timely adjustments. Conversely, low-risk patients could be managed with less aggressive approaches, focusing on minimizing treatment-related toxicity and enhancing quality of life through routine surveillance and supportive care. This model also guides monitoring protocols, as high-risk patients may require more frequent evaluations, including advanced imaging or biomarker assessments, to detect relapses early. Overall, incorporating this predictive model into routine practice not only enhances individualized patient care but also promotes a proactive approach to managing AML, ultimately aiming for improved outcomes, survival rates, and overall patient well-being.

The identification of MMP7 and F12 as critical biomarkers in this context is particularly noteworthy. MMP7, recognized for its role in extracellular matrix remodeling, has been implicated in the pathogenesis of several cancers, including AML [20–23]. In this study, MMP7 was highly expressed in acute myeloid leukemia (AML) and exhibited a significant correlation with adverse clinical outcomes. Despite its implications in AML, research specifically examining MMP7 in blood cancers remains remarkably limited, yet high levels of MMP7 have been associated with increased leukemic cell migration and resistance to apoptosis, suggesting its crucial role in disease progression and highlighting its potential as a therapeutic target [24–26]. Given the lack of comprehensive studies on MMP7 in hematological malignancies, further investigation into its role in AML and related conditions is essential to fully understand its impact on disease progression and treatment strategies.

In this study, we identified F12 as a prognostic factor through univariate Cox regression analysis. Notably, F12 expression was significantly upregulated in the C1 group, and high F12 expression correlated with poorer outcomes in Kaplan–Meier survival analysis. Numerous studies have established a correlation between elevated F12 levels and thrombosis, underscoring its pivotal role in coagulation pathways [27–29], which can create a hypoxic environment and establish a hypercoagulable state in the tumor microenvironment [11]. This process may eventually result in significant alterations to immune cell infiltration and functionality, thereby reshaping the tumor microenvironment. Such reshaping facilitates the accumulation of immunosuppressive immune cells, encourages tumor cell immune evasion, and promotes tumor cell migration, collectively contributing to tumor growth and metastasis [30, 31]. Importantly, thrombosis is linked to the reconfiguration of the tumor microenvironment, which can profoundly affect tumor progression and treatment responses. Thus, F12 may be a critical factor in both AML and other malignancies, with its role in thrombus formation carrying broader implications for tumor biology. Elevated F12 levels may not only indicate poor prognosis in AML but also signify resistance to therapies due to these immunological changes. Despite the limited literature addressing the relationship between F12 and AML prognosis, our findings suggest that F12 influences tumor progression and treatment response, emphasizing the importance of further exploring its underlying mechanisms and evaluating its potential as a therapeutic target in AML and other types of malignancies.

Despite these promising insights, this study is not without limitations. The retrospective design of this analysis could introduce biases associated with patient selection and data interpretation. The relatively small number of clinical samples in our validation cohort represents another constraint, though we are actively collaborating with multiple medical centers to expand our sample collection for future validation studies. Additionally, the absence of experimental validation restricts our ability to draw definitive conclusions regarding the functional roles of MMP7 and F12. Future studies should prioritize prospective research with expanded multicenter cohorts and functional assays to confirm these findings and investigate the underlying mechanisms of how these biomarkers influence both the tumor microenvironment and treatment responses in AML.

Moreover, while our analysis demonstrates correlations between coagulation scores and immune cell populations, it does not delve into the functional implications of these associations. Understanding the mechanisms driving these interactions is crucial, as it may uncover novel therapeutic targets for improving treatment outcomes in AML. In summary, this study highlights the critical roles of coagulation-related genes, particularly MMP7 and F12, in AML prognosis and their potential implications in therapeutic strategies. As our understanding of the complex interactions within the tumor microenvironment deepens, the development of predictive models such as the one presented here will become increasingly vital in guiding personalized treatment approaches for AML patients.

## Conclusion

In conclusion, our study reveals the significant role of coagulation-related genes in Acute Myeloid Leukemia (AML), identifying distinct molecular subtypes linked to survival outcomes. We developed a robust prognostic model integrating these gene expressions with clinical factors, effectively stratifying patient risk. The central hub biomarkers MMP7 and F12 emerged as promising therapeutic targets and prognostic indicators. These findings enhance the understanding of AML’s molecular mechanisms and contribute to personalized treatment strategies, ultimately improving patient care and outcomes.

## Methods and materials

### Data collection

RNA-seq transcriptome data for the Acute Myeloid Leukemia (AML) cohort along with relevant clinicopathological features and prognostic details were obtained from The Cancer Genome Atlas (TCGA, https://cancergenome.nih.gov/) as the training dataset, excluding cases without survival information. Gene expression data from TCGA-LAML in FPKM format were normalized using DESeq2, while microarray data from the validation set (GSE71014, containing 104 AML samples with survival annotations from GEO) were processed using the limma package. To address technical variability, we applied ComBat batch effect correction from the sva package, with correction efficacy verified through PCA visualization. Rigorous quality control measures were implemented including sample clustering analysis, expression distribution evaluation, and outlier detection based on inter-sample correlation. Clinical parameters encompassing age, gender, and cytogenetic abnormalities were incorporated as covariates for outcome analyses. The study utilized 138 coagulation-related genes curated from the Molecular Signatures Database (MSigDB) via the GSEA platform (http://www.broadinstitute.org/gsea/msigdb/) for subsequent molecular profiling.

### Consensus clustering analysis

We performed unsupervised consensus clustering on coagulation-related genes to identify distinct molecular subtypes within the dataset, validating subtype differentiation through principal component analysis (PCA) to visualize cluster separation. The optimal number of clusters was determined using the R package “ConsensusClusterPlus,” performing 100 iterations with a resampling rate of 0.8 (pItem = 0.8) to assess cluster stability. After testing various cluster numbers, we established that the optimal clustering effect occurred with a *k* value of 2, indicating a clear separation between the two subgroups.

To evaluate clinical relevance, we performed Kaplan–Meier survival analysis to estimate overall survival (OS) differences between identified subgroups, employing the log-rank test to statistically assess significance. This analysis provided insights into how coagulation-related gene expression profiles correlate with prognostic outcomes in AML patients.

### Identification of differentially expressed genes (DEGs) between clusters and enrichment analysis

We conducted comprehensive enrichment analyses of DEGs identified between clusters, utilizing Gene Ontology (GO), Kyoto Encyclopedia of Genes and Genomes (KEGG), and Gene Set Enrichment Analysis (GSEA) via the R package “clusterProfiler.” GO functional annotation was categorized into Biological Process (BP), Cellular Component (CC), and Molecular Function (MF), elucidating the roles of these genes in various biological contexts.

For GSEA, we utilized the “c5.go.symbols.gmt” reference gene sets to investigate enriched pathways and functional categories associated with the clusters. This combination of analyses systematically characterized the biological functions, pathways, and molecular mechanisms potentially driving differences between AML subgroups.

### Tumor immune landscape analysis between different clusters

We analyzed the proportions of 22 immune cell subtypes in the TCGA cohort using the CIBERSORT (Cell-type Identification By Estimating Relative Subsets Of RNA Transcripts) algorithm, calculating ESTIMATE (Estimation of STromal and Immune cells in MAlignant Tumor tissues using Expression data) scores, immune scores, stromal scores, and tumor purity with the ESTIMATE algorithm to explore the tumor immune microenvironment (TIME) across clusters. The CIBERSORT algorithm was configured with 1000 permutations for accurate estimation of immune cell proportions. One-way ANOVA was conducted to assess expression differences of Human Leukocyte Antigen (HLA) proteins and immune checkpoint molecules among clusters.

### Construction and validation of prognostic model based on coagulation-related genes

To develop and validate a prognostic model, we identified relevant genes shared between the TCGA-LAML and GSE71014 datasets. Batch correction was performed to adjust for technical differences in gene expression profiles. We extracted gene expression levels related to coagulation for further analysis.

Univariate Cox regression analysis identified prognostic genes significantly associated with OS in AML patients. Lasso regression was then applied to construct a refined risk score formula based on relevant coagulation-related genes, stratifying patients into high-risk and low-risk groups based on median risk score as the cutoff point.

Kaplan–Meier survival analysis was performed to compare OS between the risk groups, and risk score distribution plots were generated to illustrate patient stratification, assessing the model’s prognostic power across multiple datasets.

### Identification of hub coagulation-related gene using machine learning techniques

To identify central hub genes for AML prognosis and treatment, we employed both random forest (RF) and support vector machine (SVM) algorithms, using the “e1071” and “randomForest” R packages, respectively. Initially, Lasso regression identified candidate genes associated with AML outcomes. We intersected these genes with those identified by RF and SVM, designating common genes as central hub biomarkers critical for prognosis and therapeutic targeting. This integrated approach, combining machine learning techniques and statistical methods, rigorously identified and validated key genes underlying AML’s molecular mechanisms.

### Comprehensive analysis of hub gene expression and its prognostic and immunological implications in AML

After identifying the hub genes, we conducted a comprehensive analysis to explore their roles and potential functions in AML. First, we examined correlations among the selected hub genes to uncover possible interactions and co-regulatory mechanisms, providing insights into their collective influence on AML progression. Using data from the TCGA cohort, we assessed the relationship between hub gene expression levels and patient survival outcomes. Kaplan–Meier survival analysis showed significant associations between hub gene expression and overall survival, underscoring their prognostic value in AML.

Next, we explored the relationship between hub gene expression and immune checkpoint molecules, as these interactions could contribute to immune evasion and therapy resistance. We also examined the impact of hub gene expression on response to immune checkpoint blockade (ICB) therapy, offering insights into their potential influence on treatment efficacy. To further elucidate the functional roles of these hub genes, we used single-cell RNA sequencing data to analyze their expression across various cell types in the AML microenvironment. This analysis provided a detailed view of hub gene expression heterogeneity, revealing their potential roles in specific cellular contexts and their impact on tumor biology and therapeutic responses.

### Bone marrow collection and processing for AML study

Bone marrow samples were collected from 6 newly diagnosed acute myeloid leukemia (AML) patients and 6 healthy donors between June 2023 and June 2024 at the Department of Hematology, the Affiliated Cancer Hospital of Zhengzhou University, Zhengzhou, China. These samples were specifically obtained for subsequent molecular analyses, including qPCR experiments, to investigate gene expression profiles associated with AML. The study protocol was approved by the institutional ethics committee, and written informed consent was obtained from all participants prior to sample collection. The samples were processed and stored under standardized conditions to ensure RNA integrity and suitability for downstream qPCR analysis.

### Cell line source and culture methods

We utilized the human acute myeloid leukemia SKM-1 cell line (a suspension cell line) for our experiments, which was purchased from Procell Life Science & Technology Co., Ltd (Wuhan, China). The SKM-1 cells were maintained under standard culture conditions (37 °C, 5% CO_2_) in RPMI-1640 medium supplemented with 10% fetal bovine serum (FBS) and 1% antibiotic–antimycotic solution.

### RNA extraction and qPCR gene expression analysis

RNA was isolated from fresh tissue samples with TRI Reagent (Biosharp, Beijing, China) in accordance with the manufacturer’s guidelines. The RNA concentration was quantified using a NanoDrop 2000 spectrophotometer (Thermo Scientific, Wilmington, DE, USA). Following this, 2 µg of the total RNA was converted into complementary DNA (cDNA) using a Reverse Transcription Kit (Biosharp, Beijing, China), adhering to the specified protocol. Quantitative PCR (qPCR) was conducted using SYBR Green qPCR Mix (Biosharp, Beijing, China) on a ViiA 7 Real-Time PCR system (Applied Biosystems, Foster City, CA, USA). The Ct values for the target genes were normalized against the reference gene β-Actin, and the relative expression levels were determined using the 2^−ΔΔCt^ method. The primer sequences utilized were: β-Actin (forward: 5′-CATGTACGTTGCTATCCAGGC-3′, reverse: 5′-CTCCTTAATGTCACGCACGAT-3′), F12 (forward: 5′-AACACTTTCGATTCCACCTTGG-3′, reverse: 5′-TTGTGGGTACATTTGTGGTACAG-3′), and MMP7 (forward: 5′-GAGTGAGCTACAGTGGGAACA-3′, reverse: 5′-CTATGACGCGGGAGTTTAACAT-3′).

### siRNA transfection and proliferation analysis

SKM-1 cells were transfected with 50 nM of target-specific or control siRNAs using Lipofectamine™ 3000 (Invitrogen) in 6-well plates. Two MMP7-targeting siRNAs were used: MMP7-1 (SS: 5′-CCAACAGUUUAGAAGCCAATT-3′, AS: 5′-UUGGCUUCUAAACUGUUGGTT-3′) and MMP7-2 (SS: 5′-GCAGUCUAGGGAUUAACUUTT-3′, AS: 5′-AAGUUAAUCCCUAGACUGCTT-3′), along with two F12-targeting siRNAs (F12-1: SS: 5′-GCUGGUGUGUGAGGACCAAGC-3′, AS: 5′-UUGGUCCUCACACACCAGCGG-3′; F12-2: SS: 5′-CGUGGUGCUUCGUGCUGAACC-3′, AS: 5′-UUCAGCACGAAGCACCACGGG-3′) and scrambled control siRNA (SS: 5′-UUCUCCGAACGUGUCACGUTT-3′, AS: 5′-ACGUGACACGUUCGGAGAATT-3′). Knockdown efficiency was confirmed by qRT-PCR at 24 h. For proliferation assays, transfected cells (5 × 10^3^ cells/well) were seeded in 96-well plates and analyzed at 24 h intervals (0–72 h) using CCK-8 reagent (10 μL/well, 2 h incubation), measuring absorbance at 450 nm. Untransfected and Lipofectamine-only controls were included. Data were normalized to 0 h and analyzed by two-way ANOVA with Tukey’s post-hoc test (GraphPad Prism v9.0), with six replicates across three independent experiments.

### Statistical analysis

All statistical analyses were performed using R software, version 4.4.0. Differences between two groups were assessed using the Wilcoxon rank-sum test for nonparametric data or Student’s t-test for parametric data. For comparisons involving three or more groups, one-way analysis of variance (ANOVA) was employed, with the Mann–Whitney U test serving as a nonparametric alternative. Relationships between variables were explored using Pearson’s correlation test for linear associations and Spearman’s correlation test for non-linear correlations. Survival analysis employed Kaplan–Meier curves, with survival differences assessed via the log-rank test for significance. All statistical tests were conducted as two-sided, with *p*-values less than 0.05 considered statistically significant. This comprehensive statistical framework ensured rigorous evaluation of our findings, enhancing the accuracy and interpretability of results.

## Data Availability

No datasets were generated or analysed during the current study.

## Abbreviations

AML: Acute Myeloid Leukemia
TCGA: The Cancer Genome Atlas
GEO: Gene Expression Omnibus
DEGs: Differentially Expressed Genes
ELN: European Leukemia Network
VTE: Venous Thromboembolism
ATE: Arterial Thromboembolism
TME: The tumor microenvironment
TF: Tissue Factor
NK: Natural Killer
PD-L1: Programmed cell Death protein Ligand 1
FPKM: Fragments Per Kilobase Million
TCGA-LAML: The Cancer Genome Atlas-Acute Myeloid Leukemia
MSigDB: Molecular Signatures Database
GSEA: Gene Set Enrichment Analysis
PCA: Principal Component Analysis
OS: Overall Survival
GO: Gene Ontology
KEGG: Kyoto Encyclopedia of Genes and Genomes
BP: Biological Process
CC: Cellular Component
MF: Molecular Function
CIBERSORT: Cell-type Identification By Estimating Relative Subsets Of RNA Transcripts
ESTIMATE: Estimation of STromal and Immune cells in MAlignant Tumor tissues using Expression data
HLA: Human Leukocyte Antigen
RF: Random Forest
SVM: Support Vector Machine
ICB: Immune Checkpoint Blockade
ANOVA: One-way analysis of variance
logFC: Log Fold Change
CXCR: C-X-C chemokine receptor
ECM-receptor interaction: Extracellular Matrix-receptor interaction
Tregs: Regulatory T cells
AUC: Area Under the Curve
ROC: Receiver Operating Characteristic
C-index: Concordance index
EryPro: Erythrocyte Progenitor

## References

[1] Khwaja A, Bjorkholm M, Gale RE, Levine RL, Jordan CT, Ehninger G, et al. Acute myeloid leukaemia. Nat Rev Dis Primers. 2016;2:16010. 27159408 10.1038/nrdp.2016.10

[2] Miller KD, Nogueira L, Devasia T, Mariotto AB, Yabroff KR, Jemal A, et al. Cancer treatment and survivorship statistics, 2022. CA Cancer J Clin. 2022;72(5):409–36. 35736631 10.3322/caac.21731

[3] Chen X-X, Li Z-P, Zhu J-H, Xia H-T, Zhou H. Systematic analysis of autophagy-related signature uncovers prognostic predictor for acute myeloid leukemia. DNA Cell Biol. 2020;39(9):1595–605. 32783661 10.1089/dna.2020.5667 PMC7482110

[4] Han S-Y. Small molecule induced FLT3 degradation. Pharmaceuticals. 2022;15(3):320. 35337118 10.3390/ph15030320 PMC8954439

[5] Chen S, Zhu H, Jin M, Yuan H, Liu Z, Li J, et al. Molecular and clinical characteristics of IDH mutations in Chinese NSCLC patients and potential treatment strategies. Cancer Med. 2022;11(22):4122–33. 35526267 10.1002/cam4.4764 PMC9678110

[6] Döhner H, Estey E, Grimwade D, Amadori S, Appelbaum FR, Büchner T, et al. Diagnosis and management of AML in adults: 2017 ELN recommendations from an international expert panel. Blood. 2017;129(4):424–47. 27895058 10.1182/blood-2016-08-733196 PMC5291965

[7] Blom JW, Doggen CJM, Osanto S, Rosendaal FR. Malignancies, prothrombotic mutations, and the risk of venous thrombosis. JAMA. 2005;293(6):715–22. 15701913 10.1001/jama.293.6.715

[8] Timp JF, Braekkan SK, Versteeg HH, Cannegieter SC. Epidemiology of cancer-associated venous thrombosis. Blood. 2013;122(10):1712–23. 23908465 10.1182/blood-2013-04-460121

[9] Mulder FI, Horváth-Puhó E, van Es N, van Laarhoven HWM, Pedersen L, Moik F, et al. Venous thromboembolism in cancer patients: a population-based cohort study. Blood. 2021;137(14):1959–69. 33171494 10.1182/blood.2020007338

[10] Adelborg K, Corraini P, Darvalics B, Frederiksen H, Ording A, Horváth-Puhó E, et al. Risk of thromboembolic and bleeding outcomes following hematological cancers: a Danish population-based cohort study. J Thromb Haemost. 2019;17(8):1305–18. 31054195 10.1111/jth.14475

[11] Lei C, Li Y, Yang H, Zhang K, Lu W, Wang N, et al. Unraveling breast cancer prognosis: a novel model based on coagulation-related genes. Front Mol Biosci. 2024;11:1394585. 38751445 10.3389/fmolb.2024.1394585 PMC11094261

[12] Gofrit SG, Shavit-Stein E. The neuro-glial coagulonome: the thrombin receptor and coagulation pathways as major players in neurological diseases. Neural Regen Res. 2019;14(12):2043–53. 31397331 10.4103/1673-5374.262568 PMC6788244

[13] Feinauer MJ, Schneider SW, Berghoff AS, Robador JR, Tehranian C, Karreman MA, et al. Local blood coagulation drives cancer cell arrest and brain metastasis in a mouse model. Blood. 2021;137(9):1219–32. 33270819 10.1182/blood.2020005710

[14] Dann R, Hadi T, Montenont E, Boytard L, Alebrahim D, Feinstein J, et al. Platelet-derived MRP-14 induces monocyte activation in patients with symptomatic peripheral artery disease. J Am Coll Cardiol. 2018;71(1):53–65. 29301628 10.1016/j.jacc.2017.10.072 PMC5882198

[15] Graf C, Wilgenbus P, Pagel S, Pott J, Marini F, Reyda S, et al. Myeloid cell-synthesized coagulation factor X dampens antitumor immunity. Sci Immunol. 2019;4(39):eaaw8405. 31541031 10.1126/sciimmunol.aaw8405 PMC6830514

[16] Wang Y, Bin T, Tang J, Xu X-J, Lin C, Lu B, et al. Construction of an acute myeloid leukemia prognostic model based on m6A-related efferocytosis-related genes. Front Immunol. 2023;14:1268090. 38077322 10.3389/fimmu.2023.1268090 PMC10704160

[17] Zhang C, Wen R, Wu G, Li G, Wu X, Guo Y, et al. Identification and validation of a prognostic risk-scoring model for AML based on m7G-associated gene clustering. Front Oncol. 2023;13:1301236. 38273850 10.3389/fonc.2023.1301236 PMC10808397

[18] Jiang G, Jin P, Xiao X, Shen J, Li R, Zhang Y, et al. Identification and validation of a novel CD8+ T cell-associated prognostic model based on ferroptosis in acute myeloid leukemia. Front Immunol. 2023;14:1149513. 37138885 10.3389/fimmu.2023.1149513 PMC10150955

[19] Wang X, Sun H, Dong Y, Huang J, Bai L, Tang Z, et al. Development and validation of a cuproptosis-related prognostic model for acute myeloid leukemia patients using machine learning with stacking. Sci Rep. 2024;14(1):2802. 38307903 10.1038/s41598-024-53306-7 PMC10837443

[20] Hu H-M, Lee H-L, Liu C-J, Hsieh Y-H, Chen Y-S, Hsueh K-C. Loss of MTA2-mediated downregulation of PTK7 inhibits hepatocellular carcinoma metastasis progression by modulating the FAK-MMP7 axis. Environ Toxicol. 2024;39(4):1897–908. 38050825 10.1002/tox.24073

[21] Fan W, Cao D, Yang B, Wang J, Li X, Kitka D, et al. Hepatic prohibitin 1 and methionine adenosyltransferase α1 defend against primary and secondary liver cancer metastasis. J Hepatol. 2024;80(3):443–53. 38086446 10.1016/j.jhep.2023.11.022 PMC10922446

[22] Ou S, Chen H, Wang H, Ye J, Liu H, Tao Y, et al. Fusobacterium nucleatum upregulates MMP7 to promote metastasis-related characteristics of colorectal cancer cell via activating MAPK(JNK)-AP1 axis. J Transl Med. 2023;21(1):704. 37814323 10.1186/s12967-023-04527-3 PMC10561506

[23] Zhu R, Tao H, Lin W, Tang L, Hu Y. Identification of an immune-related gene signature based on immunogenomic landscape analysis to predict the prognosis of adult acute myeloid leukemia patients. Front Oncol. 2020;10:574939. 33330048 10.3389/fonc.2020.574939 PMC7714942

[24] Wu Y, Pan S, Leng J, Xie T, Jamal M, Yin Q, et al. The prognostic value of matrix metalloproteinase-7 and matrix metalloproteinase-15 in acute myeloid leukemia. J Cell Biochem. 2019;120(6):10613–24. 30809850 10.1002/jcb.28351

[25] Li G, Gao Y, Li K, Lin A, Jiang Z. Genomic analysis of biomarkers related to the prognosis of acute myeloid leukemia. Oncol Lett. 2020;20(2):1824–34. 32724426 10.3892/ol.2020.11700 PMC7377096

[26] Wu J, Song Y. Expression and clinical significance of serum MMP-7 and PTEN levels in patients with acute myeloid leukemia. Oncol Lett. 2018;15(3):3447–52. 29563992 10.3892/ol.2018.7799 PMC5854936

[27] Sparkenbaugh EM, Henderson MW, Miller-Awe M, Abrams C, Ilich A, Trebak F, et al. Factor XII contributes to thrombotic complications and vaso-occlusion in sickle cell disease. Blood. 2023;141(15):1871–83. 36706361 10.1182/blood.2022017074 PMC10122107

[28] Xu P, Zhang Y, Guo J, Li H, Konrath S, Zhou P, et al. A single-domain antibody targeting factor XII inhibits both thrombosis and inflammation. Nat Commun. 2024;15(1):7898. 39266545 10.1038/s41467-024-51745-4 PMC11393108

[29] Shamanaev A, Litvak M, Ivanov I, Srivastava P, Sun M-F, Dickeson SK, et al. Factor XII structure-function relationships. Semin Thromb Hemost. 2024;50(7):937–52. 37276883 10.1055/s-0043-1769509 PMC10696136

[30] Chen Z-Q, Zuo X-L, Cai J, Zhang Y, Han G-Y, Zhang L, et al. Hypoxia-associated circPRDM4 promotes immune escape via HIF-1α regulation of PD-L1 in hepatocellular carcinoma. Exp Hematol Oncol. 2023;12(1):17. 36747292 10.1186/s40164-023-00378-2 PMC9903500

[31] Yan D, Cai S, Bai L, Du Z, Li H, Sun P, et al. Integration of immune and hypoxia gene signatures improves the prediction of radiosensitivity in breast cancer. Am J Cancer Res. 2022;12(3):1222–40. 35411250 PMC8984882

